# Safety assessment of cyanoacrylate closure for treatment of varicose veins in a large-scale national survey in Japan

**DOI:** 10.1016/j.jvsv.2024.102160

**Published:** 2024-12-18

**Authors:** Michihisa Umetsu, Masayuki Hirokawa, Eri Fukaya, Eiichi Teshima, Hitoshi Kusagawa, Toshiya Nishibe, Makoto Mo, Tomohiro Ogawa

**Affiliations:** a Division of Vascular Surgery, Department of Surgery, Tohoku University Hospital, Sendai, Japan; b Ochanomizu Vascular and Vein Clinic, Tokyo, Japan; c Division of Vascular Surgery, Stanford School of Medicine, Palo Alto, CA; d Fukuoka Wajiro Hospital, Vascular and Endovascular Surgery, Fukuoka, Japan; e Matsusaka Ohta Clinic, Matsusaka, Japan; f Department of Medical Management and Informatics, Hokkaido Information University, Ebetsu, Japan; g Department of Cardiovascular Surgery, Yokohama Minami Kyosai Hospital, Yokohama, Japan; h Department of Surgery, Yokohama City University, Yokohama, Japan; i Japanese Society of Phlebology, Tokyo, Japan; j Department of Cardiovascular Surgery, Fukushima Dai-Ichi Hospital, Fukushima, Japan; k Japanese Regulatory Committee for Endovascular Treatment of Varicose Veins, Tokyo, Japan

**Keywords:** Adverse event, Complication, Cyanoacrylate closure, National survey, Varicose vein

## Abstract

**Objective:**

Cyanoacrylate closure (CAC) is a minimally invasive technique for treating axial venous reflux. However, the incidence of serious adverse events (AEs) related to CAC is concerning. With an increasing number of patients undergoing CAC and insufficient safety data in Japan, this study aimed to investigate the safety profile of CAC, focusing on the types and incidence of AEs.

**Methods:**

A nationwide survey was conducted by the Japanese Regulatory Committee for Endovascular Treatment of Varicose Veins between November 2023 and December 2023. Data were collected from 1017 institutions, covering 24,209 patients who underwent CAC at 335 institutions between January 2020 and October 2023. Thromboembolism, phlebitis, hypersensitivity reactions, granuloma formation, infection, bleeding, death, and need for glue resection were documented as unfavorable events or outcomes.

**Results:**

Venous thromboembolism developed in 142 patients (0.59%). Pulmonary embolism, proximal deep vein thrombosis, and ablation-related thrombus extension developed in 3 (0.01%), 9 (0.04%), and 95 (0.39%) patients, respectively. Localized phlebitis that required additional treatment was observed in 1656 patients (6.8%). Of the localized hypersensitivity cases, 960 (58%) required oral antihistamines and 268 (16%) required oral and/or intravenous steroids. Furthermore, 65 patients (0.27%) developed systemic hypersensitivity that required systemic steroids. No patients developed a stroke or anaphylaxis. One patient died owing to pulmonary embolism. Glue resection was performed in nine patients with delayed infection (n = 4), hypersensitivity reactions (n = 4), or a foreign body granuloma (n = 1). The incidence of hypersensitivity reactions was similar among institutions. However, the incidence of thrombosis-related events significantly differed between the high-volume and low-volume institutions. The incidence of proximal deep vein thrombosis (0.13% vs 0.01%; *P* < .001; odds ratio, 12.5; 95% confidence interval, 2.6-60.3) and ablation-related thrombus extension (0.73% vs 0.30%; *P* < .001; odds ratio, 2.5; 95% confidence interval, 1.66-3.77) was significantly higher in low-volume institutions than in high-volume centers.

**Conclusions:**

A nationwide survey of CAC for varicose veins in Japan demonstrated that it was a safe procedure with a low rate of serious AEs, such as venous thromboembolism. However, hypersensitivity reactions requiring steroid administration and systemic allergic reactions were observed in some patients.

**Article Highlights**

• **Type of Research:** Retrospective analysis of Japanese national survey data of cyanoacrylate closure (CAC)
• **Key Findings:** A Japanese national survey included data from 24,209 patients across 1017 institutions who underwent CAC. Venous thromboembolism occurred in 142 patients (0.59%), and 1656 (6.8%) developed localized phlebitis requiring treatment. One death was reported owing to pulmonary embolism, and nine patients required glue resection for various complications.
• **Take Home Message:** CAC treatment for varicose veins is safe in Japan. However, a small number of cases required systemic steroids or resection of CAC-treated veins.

Cyanoacrylate closure (CAC) is a safe, minimally invasive, nonthermal, nontumescent treatment modality. However, adverse events (AEs) specific to CAC, including hypersensitivity, have been reported in 6% to 11% of cases.^1^^,^^2^ A recent report identified a high incidence of serious AEs associated with CAC in public regulatory databases for medical devices, including the Food and Drug Administration's Total Product Life Cycle database.^3^ This has raised concerns regarding AEs; however, without a defined total number of procedures, the true incidence of AEs remains unclear. Thus, further clarification is required using large datasets.

For CAC treatments in Japan, VenaSeal (Medtronic Inc., Minneapolis, MN) has been covered by the universal health care system since 2019. Other CAC products, such as Vena-Block, VeinOff, VariClose, and Venex, are not covered by insurance. To obtain reimbursement via insurance, providers must be trained and certified by the Japanese Regulatory Committee for Endovascular Treatment of Varicose Veins (JRCETVV). In 2020, a guideline for CAC was established by the Japanese Society of Phlebology, and compliance with the guideline is required to obtain reimbursement for the procedure.^4^ Thus, most CAC procedures in Japan are performed in accordance with the guideline, with the appropriate documents, and under the supervision of JRCETVV.

The 2020 Japanese guidelines provide indications and contraindications for CAC in addition to educational material required to use cyanoacrylate adhesives. Similar to the indications for the conventional endovenous thermal ablation (EVTA), symptomatic primary axial reflux with varicose veins is an indication for CAC. The guidelines state that a history of allergies, systemic inflammatory diseases, autoimmune diseases, and granulomatous diseases are relative contraindications for CAC. Patients with an allergic diathesis to cyanoacrylate or those who frequently use cyanoacrylate products for eyelash extensions and artificial nails implantation are also classified as patients who require attention. This is because frequent exposure to such products may trigger allergic reactions. Furthermore, given the concern that cyanoacrylate may pose a risk of infection, it is contraindicated in patients with stasis ulcers and cellulitis. Nonsteroidal anti-inflammatory drugs are recommended as the initial treatment for allergic reactions. Additional treatment with antihistamines or steroids is recommended if localized itching at the treatment area or generalized urticaria develops, depending on the severity of phlebitis symptoms. Follow-up visits and ultrasound examinations are recommended within 2 weeks and at 1 to 3 months after CAC to monitor for any AEs.^4^

Given this reporting and appropriate use structure, the JRCETVV aimed to identify participating centers from this reporting system that were obtained via a nationwide survey among CAC practitioners to identify CAC-associated AEs in Japan. The goal of this survey was to evaluate the safety profile of CAC in clinical practice in Japan by focusing on serious AEs.^5^ Data were successfully collected via the survey from 61% of the JRCETVV-registered institutions. This nationwide survey was conducted by the JRCETVV in collaboration with the Japanese Society of Phlebology.

## Methods

### Study design and data collection

The CAC AEs National Survey was a web-based survey conducted between November 2023 and December 2023 at the 1017 JRCETVV-registered institutions. The survey was sent to the CAC-accredited physician and a response was received from the physician who performed the procedure. Data from patients who had undergone a CAC between January 2020 and October 2023 were collected. Reports of serious AEs were queried individually with the reporting institutions to obtain detailed case histories, and the data were verified subsequently by the event committee (T.O., M.H., and M.M.). This study was approved by the Fukuoka Wajiro Hospital's Ethics Committee (No: 00220; approval date: November 13, 2023). This survey complies with the ethical guidelines for medical and biological research involving human subjects that was issued in April 2023 by the Ministry of Health, Labor, and Welfare in Japan.

The survey (Appendix 1, online only) included information regarding the number of CACs performed, anesthesia method used, concomitant procedures performed, and AEs that occurred during the study period. Venous thromboembolism (VTE), stroke, iatrogenic deep vein occlusion by glue, local phlebitis (including foreign body reaction and hypersensitivity), systemic hypersensitivity, anaphylaxis, severe infection, granuloma formation, glue resection, and major bleeding were considered as serious AEs in the study. Death and the need for resection of the treated veins were identified as serious outcomes.

### Definitions

VTE was defined as the presence of deep vein thrombosis (DVT), pulmonary embolism (PE), or ablation-related thrombus extension (ARTE). ARTE was defined as a nonocclusive thrombus that developed during postoperative follow-up after CAC, which extended into the deep venous system and had a cross-sectional area of >50%.

Stroke was defined as the acute onset of a neurological deficit owing to an ischemia-induced disturbance in cerebral circulation. Iatrogenic deep vein occlusion by glue was defined as a deep vein occlusion resulting from direct glue injection into the deep vein or glue flow owing to accidental catheter insertion into a deep vein.

Localized phlebitis that required additional treatment was categorized as a foreign body reaction (necessitating administration of nonsteroidal anti-inflammatory drugs) or a hypersensitivity reaction (necessitating antihistamine or oral and/or intravenous steroid administration).

Systemic hypersensitivity was defined as cases of systemic allergy requiring oral and/or intravenous steroid administration. Type I allergy caused by glue was defined as an anaphylactic reaction, which is a life-threatening hypersensitivity reaction that elicits systemic allergic symptoms and affects multiple organs.

Severe infection was defined as cases of sepsis that required hospitalization or vein resection. Granuloma formation after a foreign body reaction was defined as the formation of a lesion exhibiting erythema, swelling, and induration at the site of glue treatment months to years after CAC.

Glue resection was defined as the removal of the vein that was injected with glue (excluding glue removal at the puncture site). A high-volume center was defined as a center in the top quartile for the number of CACs performed.

### Statistical analysis

Categorical variables are presented as the number of cases and percentages, and they were compared using the χ^2^ or Fisher's exact test. Continuous variables are expressed according to their distributions as mean ± standard deviation or median with the interquartile range (IQR). All reported *P* values were two-sided. A *P* value of <.05 was considered statistically significant. All analyses were performed using JMP (version 17.2; SAS Institute, Cary, NC).

## Results

### Data collection and institution characteristics

The survey responses were collected from 645 of the 1017 CAC-accredited institutions. CAC was not being performed actively at all the institutions that returned the survey. Furthermore, some of the CAC-accredited institutions have closed. CAC was performed in 24,209 patients at 335 institutions during the study period (Table I). CAC was performed as an outpatient procedure in 173 institutions (51.6%). The most common anesthetic method used was local anesthesia (n = 320 institutions; 95.5%), followed by general anesthesia (n = 15 institutions; 4.4%) and lumbar anesthesia (n = 2 institutions; 0.6%). Only CAC was performed in 215 institutions (64.2%), whereas CAC was combined with phlebectomy in 104 institutions (31.0%) and sclerotherapy in 16 institutions (4.8%). Of the 335 institutions that performed CACs, 248 were hospitals and 87 were private clinics (Fig). The proportion of private clinics was notably high in institutions with large caseloads. The median number of CACs performed was 24 (IQR, 7-78).

**Table I:** Survey demographics and treatment modalities used

Demographics	Number or No. (%)
Total CAC cases	24,209
No. of institutions	335
Treatment modalities	
Outpatient treatment	173 (52.6)
Anesthesia	
General	15 (4.5)
Local	320 (95.5)
Lumbar	2 (0.6)
Concomitant procedure	
Phlebectomy	104 (31.0)
Sclerotherapy	16 (4.8)
None	215 (64.2)

*CAC,* Cyanoacrylate closure.

**Fig:**
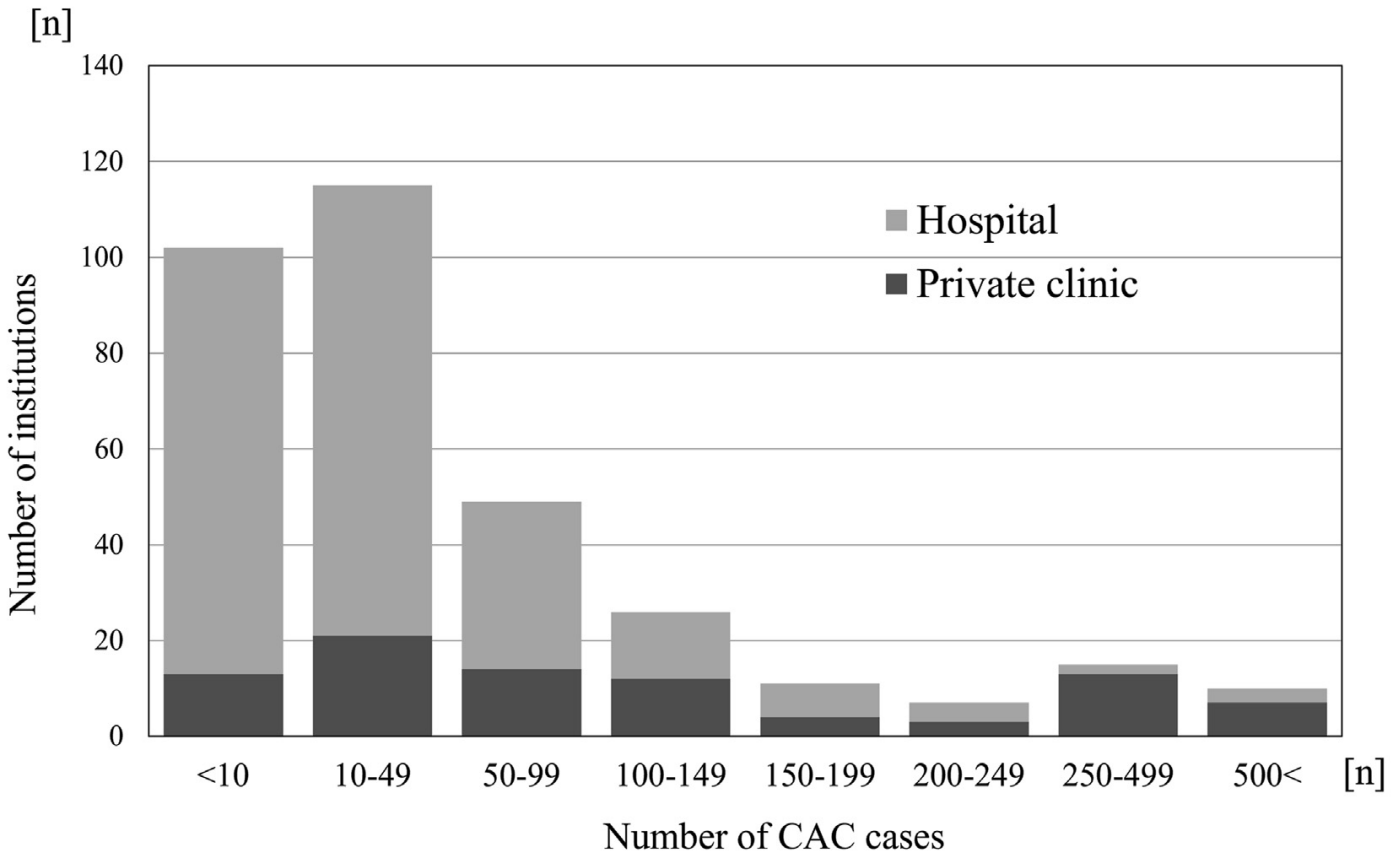
Number of CACs performed by private clinics and hospitals. Private clinics were defined as institutions without inpatient services, and hospitals were defined as institutions with inpatient services. *CAC*, Cyanoacrylate closure.

### AEs and outcomes

A summary of the AEs and outcomes of CAC is presented in Table II. VTE, including PE, proximal DVT, ARTE, and distal DVT, were reported in 142 patients (0.59%). Localized phlebitis that required additional treatment was observed in 1656 patients (6.8%), and 65 patients (0.27%) developed systemic hypersensitivity. Furthermore, four patients developed severe infections, one patient developed iatrogenic deep vein occlusion by glue, and one patient developed a granuloma. No cases of stroke or anaphylaxis were reported. One patient developed major bleeding owing to arterial bleeding at the site of concurrent phlebectomy, which required blood transfusion. Thus, the AE was unrelated to CAC. One patient died owing to PE, and nine patients required glue resection.

**Table II:** Adverse events (*AEs*) and outcomes related to cyanoacrylate closure (CAC)

Events/outcomes	No. (%)
AEs	
VTE	142 (0.59)
PE	3 (0.01)
Proximal DVT	9 (0.04)
ARTE	95 (0.39)
Distal DVT	35 (0.14)
SVT	150 (0.62)
Stroke	0
Iatrogenic deep vein occlusion by glue	1 (0.004)
Localized phlebitis requiring treatment	1656 (6.8)
Foreign body reaction	419 (25)
Hypersensitivity reaction (requiring antihistamines)	960 (58)
Hypersensitivity reaction (requiring steroids)	268 (16)
Systemic hypersensitivity	65 (0.27)
Anaphylaxis	0
Granuloma formation	1 (0.004)
Severe infection	4 (0.017)
Major bleeding	1 (0.004)
Outcomes	
Death	1 (0.004)
Glue resection	9 (0.037)

*ARTE,* Ablation-related thrombus extension; *DVT,* deep vein thrombosis; *PE,* pulmonary embolism; *SVT,* superficial venous thrombosis; *VTE,* venous thromboembolism.

### Localized phlebitis and hypersensitivity

Localized phlebitis requiring additional treatment was observed in 1656 patients (6.8%) (Table II). Foreign body reactions developed in 419 patients (1.7%), and localized hypersensitivity was observed in 1228 patients (5.1%). For the treatment of localized hypersensitivity, antihistamines were administered to 960 patients (4.0%) and oral and/or intravenous steroids were administered to 268 patients (1.1%). The median incidence of hypersensitivity reactions requiring steroids was 3.4% (IQR, 1.1%-7.1%) per institution. Furthermore, the incidence of hypersensitivity reactions requiring steroid administration and the distribution of cases per institution indicated that steroid use was higher in low-volume institutions than in high-volume centers (Appendix 2, online only).

### VTE characteristics

VTE was observed in 142 patients (0.59%; Table II). The majority of VTE cases were ARTE (n = 95 [0.39%]). PE, proximal DVT, and distal DVT were observed in 3 (0.01%), 9 (0.04%), and 35 (0.14%) patients, respectively. Superficial venous thrombosis developed in 150 patients (0.62%). Anticoagulation therapy was administered in 150 patients (51.4%) with VTE or superficial venous thrombosis.

An octogenarian woman with no known VTE risk factors died owing to PE. She underwent CAC of both small saphenous veins under local anesthesia and sedation using propofol. The total operative time was 55 minutes, and no concomitant procedures were performed. On postoperative day 11, the patient developed shortness of breath, and on day 15, she was diagnosed with PE and DVT of both lower limbs. The patient was hospitalized and treated with extracorporeal membrane oxygenation (ECMO). Anticoagulation therapy was never administered despite the absence of any contraindications for its administration prior to ECMO. She was subsequently weaned off ECMO. However, she died of pneumonia and sepsis 6 weeks after CAC.

### Glue resection

Nine patients required glue resection after CAC. Of these patients, four (0.017%) had a delayed infection, four (0.017%) had localized or systemic hypersensitivity, and one (0.004%) had a foreign body granuloma.^6^

### AEs according to experience

The differences in the incidence of AEs between high-volume and low-volume centers are shown in Table III. The high-volume centers, representing the institutions in the top quartile for number of CACs performed, were 83 institutions, in which 18,917 cases (78.1%) had been collectively performed. The incidence of phlebitis, including foreign body reaction and hypersensitivity reaction, was similar between the high-volume and low-volume institutions. The incidence of proximal DVT was significantly higher in the low-volume centers than in the high-volume centers (0.13% vs 0.01%, *P* < .001; odds ratio [OR], 12.5; and 95% confidence interval [CI], 2.6-60.3). Similarly, the incidence of ARTE was significantly higher in the low-volume centers than in the high-volume centers (0.73% vs 0.30%, *P* < .001; OR, 2.5; and 95% CI, 1.66-3.77). Furthermore, anticoagulation therapy was administered more frequently in low-volume institutions than in high-volume centers (1.1% vs 0.49%, *P* < .001; OR, 2.2; 95% CI, 1.58-3.07).

**Table III:** Comparison of the adverse events (*AEs*) between high-volume and low-volume institutions

AEs	High-volume center^a^ (n = 18,917)	Low-volume institution (n = 5292)	OR (95% CI)	*P* value
Foreign body reaction	324 (1.7)	95 (1.8)	1.05 (0.83-1.32)	.68
Hypersensitivity reactions (requiring antihistamines)	763 (4.0)	197 (3.7)	0.92 (0.78-1.08)	.31
Hypersensitivity reactions (requiring steroids)	198 (1.0)	70 (1.3)	1.27 (0.96-1.67)	.09
Systemic hypersensitivity	53 (0.28)	12 (0.23)	0.81 (0.43-1.51)	.51
PE	1 (0.01)	2 (0.04)	7.15 (0.64-78.9)	.12^b^
Proximal DVT	2 (0.01)	7 (0.13)	12.5 (2.6-60.3)	<.001^b^
ARTE	56 (0.30)	39 (0.73)	2.5 (1.66-3.77)	<.001
Requirement for anticoagulation	93 (0.49)	57 (1.1)	2.2 (1.58-3.07)	<.001

*ARTE,* Ablation-related thrombus extension; *CAC,* cyanoacrylate closure; *CI,* confidence interval; *DVT,* deep vein thrombosis; *OR,* odds ratio; *PE,* pulmonary embolism.

Data expressed as number (%).

a Institutions in the top quartile for number of CACs performed.

b Fisher's exact test used.

## Discussion

In this study, the CAC-associated AEs were extracted from a large-scale national survey of 24,209 cases, which represents 73% of all the CACs performed in Japan. The CAC was safe in most patients. However, hypersensitivity complications and other AEs were observed in a small number of patients.

CAC can be a less invasive treatment modality than EVTA. Furthermore, EVTA requires tumescent local anesthesia, which can be painful and carries the risk of ecchymosis. Additionally, there are risks of nerve injury and skin burns with EVTA, which can be avoided with CAC. However, hypersensitivity to glue is the most common AE in CAC that does not occur with EVTA. Approximately 6.8% of our study patients experienced phlebitis that required additional treatment. Most of the reactions observed in our study population seemed to be transient, although a few were prolonged. The incidence of hypersensitivity reactions requiring systemic steroids varied among the institutions in this study, ranging from 0% to 50%. Some institutions treated >20% of their patients with steroids, potentially causing an overestimation of the hypersensitivity rates. Because we defined the diagnosis and severity of hypersensitivity on the basis of medications administered, notable variations were observed between the institutions. This indicates that the reported incidence of AEs may represent a maximum estimate, because some patients received medications prophylactically, potentially inflating the reported rates without necessarily indicating true hypersensitivity.

One death case was reported in this survey. CAC was completed without any complications in the patient. Furthermore, although the association of PE with endovenous treatment could not be ruled out, the patient was at risk of developing VTE unrelated to CAC. She remained largely immobile for >1 week after the procedure for unknown reasons. Postoperative patient education should include information regarding physical activity. This case was reported to the Total Product Life Cycle.^7^

VTE can develop after CAC as well as EVTA. In the present study, proximal DVT and PE developed in 0.037% and 0.012% of the patients, respectively. ARTE developed in 0.39% of the patients. ARTE was less common in high-volume centers than in low-volume institutions, suggesting a possible association between its incidence and the learning curve. The incidence of DVT was 1.3% in a real-world postmarket evaluation in Singapore.^8^ Although our study was a retrospective observational study, the incidence of thrombosis events was not higher than that in other studies.^8^^,^^9^ It is recommended that CAC be performed ≥5 cm away from the saphenofemoral junction (SFJ). Even at this distance, the postprocedure SFJ stump reportedly measures 1.7 cm owing to the accumulation of cyanoacrylate.^10^ Thus, this distance should be maintained to prevent the development of proximal VTE. Most ARTEs are reportedly not associated with clot extension or migration, and ARTEs associated with SFJ are rare.^11^ In our study results, thrombotic events were more common in less experienced institutions than in more experienced institutions, suggesting that VTE events may decrease as experience increases. In a Japanese endovenous laser ablation survey that included 43,203 patients, classes 2, 3, and 4 endovenous heat-induced thromboses were observed in 0.73%, 0.12%, and 0.016% of the patients, respectively. Furthermore, PE developed in 0.0069% of the patients.^12^ Although the CAC-associated VTE incidence was slightly higher in this study, it seemed to be in the same range. Despite the serious AEs associated with CAC, their overall rates in Japan are generally low. In the present study, we defined ARTE as a nonocclusive thrombus that developed during postoperative follow-up after CAC, extended into the deep venous system, and had a cross-sectional area of >50%, which corresponds with EHIT class 3. We did not collect data on thrombosis with <50% extent or on cases that did not progress further. Consequently, it is possible that AEs related to thrombosis that did not require additional treatment may have been under-reported.

Foreign body granulomas are a rare complication that can only be confirmed via a large-scale study. The formation of a foreign body granuloma is hypothesized to result from the involvement of the innate immune system, including giant cells, as well as the adaptive immune system, which includes T cells and macrophages.^13^

In Japan, an updated endovascular treatment guideline for varicose veins was published in 2019, which was followed by the publication of CAC-specific guidelines in 2020.^4^^,^^14^ These guidelines clearly state that CAC can pose a risk for infection. Thus, it should not be performed in patients with venous leg ulcers complicated by an infection because a foreign body is left in situ after CAC. However, in a multicenter retrospective study that compared CAC with radiofrequency ablation in 119 patients with CEAP C6, O'Banion et al^15^ found that CAC was associated with a significantly shorter time to ulcer healing and no increased risk of infection. There is a possibility that treatment adaptations to the risk of infection may be lessened in the future. CAC is also relatively contraindicated in patients predisposed to allergic reactions or those with a history of granulomatous disease. The Japanese guidelines in addition to the need for certification by the JRCETVV to perform CAC may have influenced patient selection and prevented excessive treatment.

### Study limitations

This study has several limitations. First, although the survey was based on data extracted from medical records, it relied on memory recall by the respondents. As a result, mild cases of ARTE and phlebitis that did not require medical treatment were excluded from the study. Therefore, the incidence of complications is likely to have been underestimated. Additionally, there was no external audit of medical records to verify the accuracy or completeness of the reported complications, which may have introduced further bias. Second, information regarding the background and comorbidities of patients was lacking. Thus, the risk of AEs associated with comorbidities was not assessed. Third, because this was a retrospective survey, some AEs may not have been recorded. However, given the large sample size with an estimated registration rate of 73%, the national incidence of AEs may be considered highly accurate. Fourth, a patient selection bias may have been introduced because the indications of varicose vein treatment may differ between institutions. The degree of this limitation is likely small because this study was not designed to compare the superiority of treatments, but was designed to determine the incidence of real-world complications. Fifth, the severity of hypersensitivity reaction is unclear because data regarding the dosage and duration of steroid administration were not collected. Some institutions appeared to administer steroids at a lower threshold. Finally, the current study did not assess the proportion of CAC relative to total endovenous treatments at the participating institutions. Thus, the specific distribution at each institution remains unknown. Some institutions performed CAC in nearly all patients shortly after they received approval.

## Conclusions

The majority of patients undergoing CAC were treated safely and without AEs. However, 1.1% of the patients experienced local phlebitis that required steroid administration, and 0.27% of the patients developed systemic hypersensitivity reactions that required steroid administration. While CAC would not replace EVTA for saphenous veins, it can be optimized for treatment in selected patients.

## Author Contributions

Conception and design: MU, MM, TO

Analysis and interpretation: MU, MH, EF, ET, HK, TN, MM, TO

Data collection: MU, MH, ET, HK, TN, MM, TO

Writing the article: MU, MH, EF, HK, TN, MM

Critical revision of the article: MU, MH, EF, ET, HK, TN, MM, TO

Final approval of the article: MU, MH, EF, ET, HK, TN, MM, TO

Statistical analysis: MU

Obtained funding: Not applicable

Overall responsibility: MU, MM, TO

## Funding

This work was funded by the Japanese Society of Phlebology (JSP). JSP has no involvement in the study design or collection, analysis, and interpretation of data. JSP paid he necessary fees for writing the manuscript. JSP was not involved in the decision to submit the manuscript for publication.

## Disclosures

M.H. is a consultant for Integral Corporation.

## Appendix

Additional material for this article may be found online at www.jvsvenous.org.
