# Supplementary material for: Safety assessment of cyanoacrylate closure for treatment of varicose veins in a large-scale national survey in Japan
Source: J Vasc Surg Venous Lymphat Disord. 2024 Dec 18;13(2):102160. doi: 10.1016/j.jvsv.2024.102160 (PMC12014294; doi:10.1016/j.jvsv.2024.102160)
Supplement: Appendix 1 and 2 (online only) [file mmc1.docx]

**Supplementary Materials**

**Supplementary Appendix 1: Questionnaire about cyanoacrylate closure (CAC) treatments**

**I. Information of institution**

Institution _________________________________

Name______________________________________

Q1 Do you provide CAC treatment? (Y/N)

Q2 How many CAC treatments were performed until October 31, 2023?

Q3 Select the concomitant procedure for CAC treatment.

( )None, ( )Varicotomy, ( ) Sclerotherapy

Q4 Select the main anesthesia method for CAC treatment.

( ) Local anesthesia, ( ) Local anesthesia + intravenous anesthesia, ( ) General anesthesia

Q5 Do you provide CAC treatment mainly as inpatient or outpatient?

( ) Inpatient, ( ) outpatient

**II. Information of adverse events**

Indicate the number of cases of each adverse event.

Q6 Proximal deep vein thrombosis (DVT): Indicate the number of patients of proximal DVT, EHIT Class 4 (thrombus occluding a deep vein).

Q7 Distal DVT: Indicate the number of patients of distal DVT.

Q8 EGIT: Enter the number of patients of thrombosis into the deep venous system, with an extension into the deep system of a cross-sectional area of > 50% after CAC.

Q9 Superficial vein thrombosis: Enter the number of patients of superficial venous thrombosis, including thrombosis of varicose veins outside the treated area.

Q10 Anticoagulation: Indicate the number of patients treated with anticoagulation due to thromboembolism.

Q11 Pulmonary thromboembolism: Indicate the number of cases of pulmonary thromboembolism.

Q12 Stroke: Indicate the number of cases of stroke that occurred after CAC treatment and for which a causal relationship to the treatment could not be ruled out.

Q13 Deep vein occlusion by cyanoacrylate: Indicate the number of cases which incorrectly inserted into deep veins and occluded with cyanoacrylate.

Q14 Infection: Indicate the number of patients requiring glue resection or hospitalization for suspected infection such as puncture site infection, pyogenic cellulitis, or sepsis.

Q15 Granuloma: Indicate the number of patients with granuloma formation.

Q16 Bleeding complications: Indicate the number of patients with bleeding complications, such as extensive bleeding or pseudoaneurysms, except for minor subcutaneous bleeding.

Q17 Anaphylaxis: Indicate the number of patients resulting in anaphylaxis.

Q18 Localized Phlebitis: Indicate the number of patients requiring additional treatment due to redness, swelling, or pain at the CAC treatment site.

Q19 NSAIDs: Q18 of cases treated only with non-steroidal anti-inflammatory drug administration. (Excludes cases using antihistamines, oral steroids, and intravenous infusions）

Q20 Antihistamines: Q18 of cases treated with antihistamines. (Excludes cases using oral steroids, and intravenous infusions）

Q21 Steroids: Q18 of cases treated with oral steroids or intravenous infusions. (Excluding steroid ointment treatment)

Q22 Systemic hypersensitivity: Indicate the number of patients requiring steroid treatment. (Excluding steroid ointment treatment)

Q23 Neuropathy: Indicate the number of patients resulting in peripheral neuropathy such as abnormal skin sensation or numbness.

Q24 Other adverse events: Describe any other complications and number of cases.

**III. Outcomes**

Q25 Death: Indicate the number of deaths.

Q26 Indicate cause of death.

Q27 Indicate the number of cases with sequelae.

Q28 Indicate details of the sequelae.

Q29 Glue resection: Indicate the number of cases of glue resection due to complications.

Q30 Infection: in Q29, indicate the number of patients in which glue resection was performed due to infection.

Q31 Phlebitis: in Q29, indicate the number of patients in which glue resection was performed due to phlebitis.

Q32 Granuloma: in Q29, indicate the number of patients in which glue resection was performed due to granuloma formation.

Q33 Others: in Q29, indicate the number of patients in which glue resection was performed due to granuloma other reasons.

Q34 Free to describe any other information.

**Supplementary Appendix 2: the incidence of localized hypersensitivity required steroid administration according to institutions.**

The vertical axis shows the incidence of hypersensitivity requiring steroid administration and the horizontal axis shows the number of CAC cases per institution.


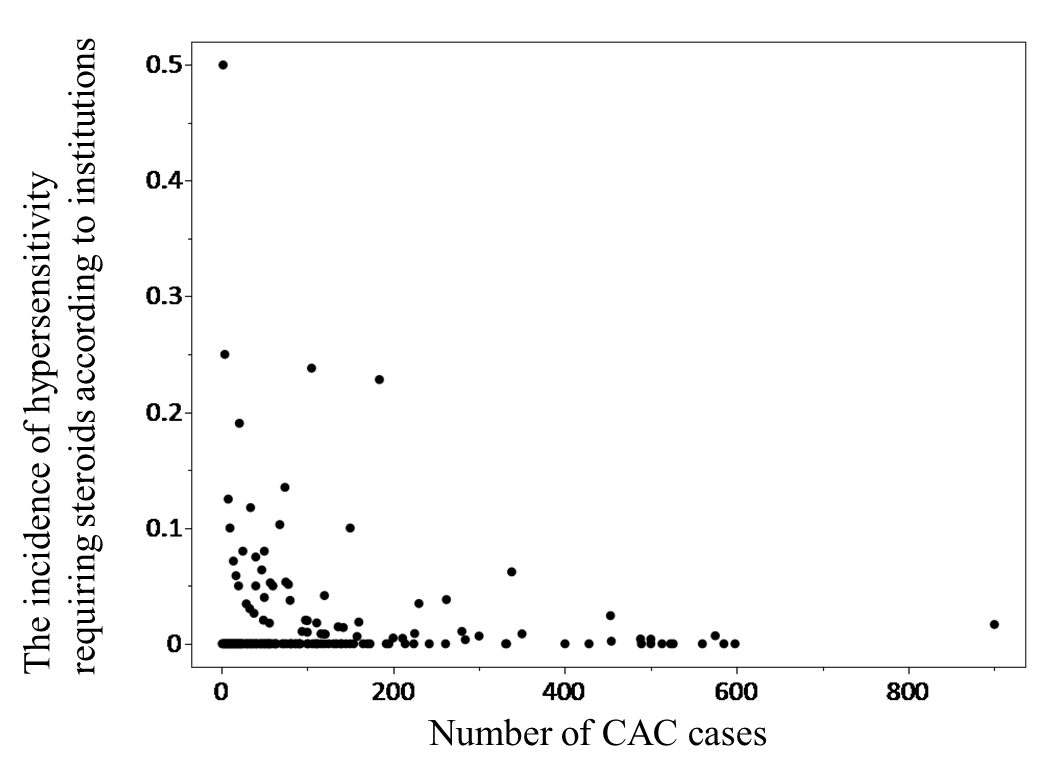


**Supplementary Appendix 3: Participating Institutions**

Fukuoka Wajiro Hospital, Takadanobaba Hospital, Osato Clinic, Sapporo Medical University, Saitama Prefectural Cardiovascular and Respiratory Disease Center, Kita-Aoyama D. Clinic, Hirosaki Central Hospital, Saiseikai Yamaguchi General Hospital, Sakae Vascular and Cardiovascular Clinic, Yamaguchi University Hospital, Suzuki Medical Clinic, Shizuoka Saiseikai General Hospital, Nanbu Tokushukai Hospital, Oji General Hospital, Nagoya Kyoritsu Hospital, Kawasaki Medical School, Mito Red Cross Hospital, Yokohama Vascular Clinic, Hiroshima Hakushima Hospital, Aiseikai Aisei Hospital, Noguchi Vascular Surgery Clinic, Osaka Red Cross Hospital, Obana Cardiovascular Clinic, Takanohashi Central Hospital, Imakiire General Hospital, Yotsuya Vascular Clinic, Ikeda Hospital, Shimada General Hospital, Ochanomizu Vascular Surgery Clinic, Iizuka Municipal Hospital, Kougame Clinic, Miharashiromachi Hospital, Akita Vascular Surgery Clinic, Kumamoto Rehabilitation Hospital, St. Mary’s Hospital, Kyushu Medical Center, JA Yamaguchi Koseiren Shuto General Hospital, Ishigakijima Tokushukai Hospital, Sapporo Teishinkai Hospital, Kansai Medical University Hospital, Niina Tsurumachi Clinic, Fujisawa Cardiovascular Clinic, Toyonaka Municipal Hospital, Kasaoka Daiichi Hospital, Yamagata Saisei Hospital, Ome Municipal General Medical Center, Saiseikai Karatsu Hospital, Isesaki Municipal Hospital, Kanayama Ekimae Heart and Vascular Clinic Kaneko Clinic, Mito Medical Center, Fukushima Red Cross Hospital, Saku Central Hospital, Yamaguchi Saiseikai Shimonoseki General Hospital, Nissan Tamagawa Hospital, Umeda Vascular Surgery Clinic, Sasebo Central Hospital, Aichi Medical Center Nagoya First Hospital, Saitama Municipal Hospital, Goto Internal Medicine Clinic, Chiba Varicose Vein and Cardiovascular Clinic, Aizu Chuo Hospital, Saga Prefectural Medical Center Koseikan, Matsusaka Ohta Clinic, International University of Health and Welfare Narita Hospital, Tokokai Kyoritsu Hospital, Ako Central Hospital, Morimoto Clinic, Jyuzen Memorial Hospital, Omori Clinic, Saitama Red Cross Hospital, Fujimoto Clinic, Maesawa Surgical Internal Medicine Clinic, Niigata Municipal Hospital, Shokoku Shintaro Clinic, Unosawa Clinic, Iizuka Hospital, Osaka City General Medical Center, Aishin Memorial Hospital, Mito Saiseikai General Hospital, Yokosuka Kyosai Hospital, Kitasenju Varicose Vein Clinic, Osaka Police Hospital, Suzuka Clinic, Maniwa Clinic, Aki Cardiovascular Surgery Clinic, Toyota Memorial Hospital, Ota Memorial Hospital, Sapporo Kohjin-kai Memorial Hospital, Nara Prefectural Seiwa Medical Center, Nishinomiya Watanabe Heart and Brain Vascular Center, Yamamoto Heart and Vascular Clinic, Kobe Rosai Hospital, Aichi Heart Clinic, Kindai University Nara Hospital, Kobe Varicose Vein Clinic, Saiseikai Kazo Hospital, Saitama Varicose Vein Clinic, Tokyo Women's Medical University, Osaka Habikino Medical Center, Aichi Medical University, Tokyo Women's Medical University Adachi Medical Center, Yūkari Vascular Clinic, Natori Dermatology Kumano-do Internal Medicine Varicose Vein Clinic, Kagaya Masa Clinic, Sagamihara Kyodo Hospital, Kamiooka Varicose Vein Clinic, Funabashi Varicose Vein Clinic, Nagahama Municipal Hospital, Shimura Dermatology Clinic, Medical Corporation Kenjinkai, Joyo Ejiri Hospital, Ureshino Medical Center, Osaka Rosai Hospital, Koto Memorial Hospital, Kasukabe Central General Hospital, Sakura Vascular Disease Clinic, Omuta Municipal Hospital, Nippon Medical School Chiba Hokusoh Hospital, Yamato Seiwa Hospital, Saitama Eastern Cardiovascular Hospital, Takai Hospital, Seikoukai Hito Hospital, Meguro Surgery, Shonan Fujisawa Tokushukai Hospital, Toho University Medical Center Sakura Hospital, Kanazawa Medical University, Mitsubishi Kyoto Hospital, Narita Red Cross Hospital, Tokyo Saiseikai Central Hospital, Onomichi Municipal Citizen Hospital, Daido Hospital, Tsukuba Medical Center Hospital, Kusatsu Heart Center, Kure Kyosai Hospital, Yamato Takada Municipal Hospital, Aomori Rosai Hospital, Osaka Saiseikai Noe Hospital, Aiiku Hospital, Fukui Saiseikai Hospital, JR Tokyo General Hospital, Iwata Vascular Surgery Clinic, Kitakanto Cardiovascular Hospital, Shinkawabashi General Hospital, Okinawa Kyodo Hospital, Nihon Seimei Hospital, Yutaka Clinic, Tokyo Kita Medical Center, Makiminato Central Hospital, Chutoen General Medical Center, Sugawara Internal Medicine and Surgery Varicose Vein Clinic, Saiseikai Wakayama Hospital, Oyama Memorial Hospital, Tottori Prefectural Kosei Hospital, Jichi Medical University Hospital, Suwa Central Hospital, Yokohama General Hospital, Kinan Hospital, Asahikawa Medical University, Yokohama Minami Kyosai Hospital, Hakodate Municipal Hospital, Fukuoka Higashi Medical Center, Juntendo University Faculty of Medicine Urayasu Hospital, Nagasaki Vascular Surgery Clinic, Ofuna Varicose Vein Clinic, Kuwana City General Medical Center, Nanba Sakamoto Surgery Clinic, Osaka Minami Medical Center, IMS Fujimi General Hospital, Imamura Hospital, Kurume University Medical Center, Nagatsuta Family Clinic, Dokkyo Medical University, Tōmei Atsugi Hospital, Fujita Medical University Bantane Hospital, Shintokyo Hospital, Kyushu Central Hospital, Ageo Central General Hospital, Fukuoka Sanno Hospital, Matsuyama Madonna Hospital, Saiseikai Niigata Hospital, Obari General Hospital, Kashiwa Kosei General Hospital, Sano Municipal Hospital, International University of Health and Welfare Mita Hospital, Sakurabashi Watanabe Hospital, Chikugo Municipal Hospital, Tonan Hospital, Dokkyo Medical University Nikko Medical Center, Fuji Podiatric & Cardiovascular Surgery Clinic, Saiseikai Izuo Hospital, Kobe City Medical Center General Hospital, Fujimori Hospital, Tanaka Clinic, Takasaki General Medical Center, Yamamoto Varicose Vein Clinic, Anjo Kosei Hospital, Iida Hospital, Nitto Hospital, Toda Central General Hospital, Koseikai Takeda Hospital, Jichi Medical University Saitama Medical Center, Saiseikai Yahata General Hospital, Nishinomiya Watanabe Heart and Brain Vascular Center, Edogawa Hospital, Kimura Internal Medicine and Surgery Clinic, Kanto Rosai Hospital, Oita Prefectural Hospital, Toyokawa City Hospital, Tokai Hospital, Omihachiman City Medical Center, Second Kawasaki Saiwai Clinic, Nishinokyo Hospital, Fukushima First Hospital, Noda Ophthalmology and Vascular Clinic, Suwa Red Cross Hospital, Juntendo Shizuoka Hospital, Jichi Medical University, Konishi Internal Medicine and Cardiovascular Clinic, Osaki City Hospital, Imazu Surgical Clinic, Sapporo Tokushukai Hospital, Shuwa General Hospital, Misato Chuo General Hospital, Shonan Fujisawa Cardiovascular Clinic, Yokkaichi City Hospital, Oda Clinic, Shonai Amarume Hospital, Sakakibara Memorial Hospital, Atsugi City Hospital, Hamamatsu University School of Medicine, Nagoya City University East Medical Center, Moriyama Mizuno Heart Clinic, Setagaya Senganwa AT Clinic, Omi Medical Center, Tsukuba Vascular Center, Cardiovascular Center Kanazawa Cardiovascular Hospital, Kyushu University Hospital, Mie Heart Center, IMS Tokyo Katsushika General Hospital, Shin Suma Hospital Surgery, Haruyama Clinic, Yokosuka City Uwamachi Hospital, Gunma University Hospital, Ikebukuro Vascular Surgery Clinic, Oita Cardiovascular Hospital, Omiya Varicose Vein Clinic, Sol Clinic Vascular Care Komagome, Kanto Central Hospital, Moriyama Memorial Hospital, Takeda General Hospital, Okayama Red Cross Hospital, Ishikawa Hospital, Yotsuba Cardiovascular Clinic, Tokyo Medical University, Yokohama Sakae Kyosai Hospital, Sunagawa City Hospital, Shinhidaka Town Shizunai Hospital, Toyooka Hospital, Himeji First Hospital, Nishiarai Hospital, Shinkuki General Hospital, Idoguchi Family Clinic, Tokyo Medical University Hachioji Medical Center, Tsuchiura Kyodo Hospital, Yoshida Clinic, Varicose Vein Clinic Yokohama, Self-Defence forces Central Hospital, Toyama University Hospital, Hyogo Medical University, Taka Clinic, Tamori Clinic, JCHO Hitoyoshi Medical Center, Kashima Heart Clinic, Kyoto Renaiss Hospital, Sogo Minamitohoku Hospital, Mizonokuchi Keiyu Clinic, Hitachi General Hospital, Ukai Family Clinic, Asakusabashi Heart and Vascular Clinic, Sakura Momiji Clinic, Chiba Varicose Vein Clinic, Ronenbyo Hospital, Soka City Hospital, Keiju General Hospital, Mikami Vascular and Varicose Vein Clinic, Ishii Hospital, JCHO Yokohama Central Hospital, Kansai Medical University General Medical Center, Naniwa Ikuno Hospital, Sato Internal Medicine, Nirasaki City Hospital, Toho Uniersity Ohashi Hospital, Hanaoka Seishu Memorial Hospital, Seirei Hamamatsu Hospital, KKR Sapporo Medical Center, Gunma Prefectural Cardiovascular Center, Yonemori Hospital, Osaka Varicose Vein Clinic, Sagamihara Kyodo Hospital Cardiology, Otsu Red Cross Hospital, Midorigaoka Hospital, Fujisawa Garden Clinic, Kawasaki Tsurumi Vascular Surgery Clinic, Kyoto Okamoto Memorial Hospital, Tokyo Vascular Surgery Clinic, Mori Clinic, Morinomiya Hospital, IMS Katsushika Heart Center, Wakayama Medical University, Minami Seikyo Hospital, Kumamoto Kinoh Hospital, Seirei Yokohama Hospital, Kumamoto Central Hospital, Matsuyama City Hospital, Koseikan, Omote Internal Medicine Diabetes Clinic, Seikeikai Hospital, Kyoto Katsura Hospital, Koga Red Cross Hospital, Nomoto Dermatology Clinic, Takeuchi Varicose Vein Clinic, Keishukai Hamada Clinic, Rakuwakai Otowa Hospital, Fukuoka City Hospital, National Hospital Organization Tokyo Medical Center, Okazaki Foot and Vascular Pain Clinic, Tokyo Vein Clinic, Hikone City Hospital, Shonan Hiratsuka Varicose Vein Clinic, Hyodo Internal Medicine Ophthalmology Heart Clinic, Morisue Clinic, Gifu Heart Center, Kawasaki City Kawasaki Hospital, Seijunkai Mito Hospital, Hiroshima Varicose Vein Clinic,
